# Digital phenotyping: towards replicable findings with comprehensive assessments and integrative models in bipolar disorders

**DOI:** 10.1186/s40345-020-00210-4

**Published:** 2020-11-17

**Authors:** Ulrich W. Ebner-Priemer, Esther Mühlbauer, Andreas B. Neubauer, Holger Hill, Fabrice Beier, Philip S. Santangelo, Philipp Ritter, Nikolaus Kleindienst, Michael Bauer, Florian Schmiedek, Emanuel Severus

**Affiliations:** 1 Mental mHealth Lab, Institute of Sport and Sport Sciences, Karlsruhe Institute of Technology, Karlsruhe, Germany; 2 Department of Psychiatry and Psychotherapy, Central Institute of Mental Health, Mannheim/Medical Faculty Mannheim, Heidelberg University, Mannheim, Germany; 3 Department of Psychiatry and Psychotherapy, University Hospital Carl Gustav Carus, Technische Universität Dresden, Dresden, Germany; 4 DIPF - Leibniz Institute for Research and Information in Education, Frankfurt, Germany; 5 Institute of Psychiatric and Psychosomatic Psychotherapy, Central Institute of Mental Health, Mannheim / Medical Faculty Mannheim, Heidelberg University, Mannheim, Germany; 6 Department of Psychology, Goethe University, Frankfurt, Germany

**Keywords:** Bipolar disorders, Digital phenotyping, Mobile sensing, Ambulatory assessment, Smartphone sensing

## Abstract

**Background:**

Digital phenotyping promises to unobtrusively obtaining a continuous and objective input of symptomatology from patients’ daily lives. The prime example are bipolar disorders, as smartphone parameters directly reflect bipolar symptomatology. Empirical studies, however, have yielded inconsistent findings. We believe that three main shortcomings have to be addressed to fully leverage the potential of digital phenotyping: short assessment periods, rare outcome assessments, and an extreme fragmentation of parameters without an integrative analytical strategy.

**Methods:**

To demonstrate how to overcome these shortcomings, we conducted frequent (biweekly) dimensional and categorical expert ratings and daily self-ratings over an extensive assessment period (12 months) in 29 patients with bipolar disorder. Digital phenotypes were monitored continuously. As an integrative analytical strategy, we used structural equation modelling to build latent psychopathological outcomes (mania, depression) and latent digital phenotype predictors (sleep, activity, communicativeness).

**Outcomes:**

Combining gold-standard categorical expert ratings with dimensional self and expert ratings resulted in two latent outcomes (mania and depression) with statistically meaningful factor loadings that dynamically varied over 299 days. Latent digital phenotypes of sleep and activity were associated with same-day latent manic psychopathology, suggesting that psychopathological alterations in bipolar disorders relate to domains (latent variables of sleep and activity) and not only to specific behaviors (such as the number of declined incoming calls). The identification of latent psychopathological outcomes that dimensionally vary on a daily basis will enable to empirically determine which combination of digital phenotypes at which days prior to an upcoming episode are viable as digital prodromal predictors.

Recently, digital phenotyping has drawn a great deal of attention in highly ranked journals (Tost et al. 2019; Raballo 2018; Jain et al. 2015; Insel 2018), as it promises objective and continuous assessments of symptomatology in patients’ daily lives (Ebner-Priemer and Santangelo 2020; Trull and Ebner-Priemer 2013). According to some authors, digital phenotyping has even the potential to outperform neuroscience and genetics (Insel 2018), which is in line with recent claims of the WHO (World Health Organization 2019) that mobile technology is the most promising way to reduce the global mental health burden. In this context, the standard prime example are bipolar disorders (BD), as parameters assessed via smartphone directly reflect bipolar symptomatology as defined in authoritative diagnostic manuals such as the DSM-5 (American Psychiatric Association 2013). For example, being more active than usual or talking quickly and loudly about many different things, both classification criteria for BD, can directly be monitored using the GPS sensors of the smartphone or microphone logs.

In stark contrast to these high expectations, a careful look into the literature (Rohani et al. 2018) reveals substantial inconsistencies. Nonreplicable findings seem to be the rule rather than the exception. Before going into detail, we want to reconsider the main concept behind digital phenotyping, which is obtaining information on psychopathological status by pure mobile sensing. To decide which smartphone parameters are informative, we need two sources of information, namely, the smartphone parameter (typically considered the predictor) and the psychopathological status/the episode (outcome). Both sources must be monitored over time to reveal meaningful variance (to “catch episodes”). In our view, the reported inconsistencies can be attributed to three main reasons: short study periods, rare outcome assessments, and an extreme fragmentation of parameters.

Searching the literature for digital phenotyping studies in BD resulted in 12 weeks as the most often used study period (see Additional file 1). Study periods that are too short are problematic. However, how can we decide which duration is appropriate? In research on BD, this is quite simple. What is needed is within-subject variance in the psychopathological status because if patients are euthymic all the time, digital phenotypes cannot predict new episodes. Expecting new episodes within 12 weeks is quite gullible, but also the more advanced approaches–6-month assessment periods (Faurholt-Jepsen et al. 2015a)—are limited. As they reported (Faurholt-Jepsen et al. 2015b) 29 years as mean age in the BD group, a clinical history of four depressive and three manic episodes, the estimated chance to experience at least one illness episode in a 6-month monitoring period is less than 40% per patient, given an estimated disorder onset age of 20 years.

Rare outcome assessments further aggravate the reported issue. In the literature (see Additional file 1), monthly clinical ratings were the most common assessment frequency. However, interviewing patients monthly about their depressive symptomatology using, e.g., the Montgomery-Asberg Depression Rating Scale (MADRS), does not provide continuous information over the entire month. It results in one depression score per month covering the previous four days; hence, six outcome assessments in a six-month study cover just 24 days. Symptomatology for the other 158 days is unknown. We illustrated this issue using our own data. Figure 1a depicts dimensional expert ratings covering the previous (in our data) three days over a 6-months study period. For approximately 85% of all days, no information on psychopathological status is available. Such a data set is limited in detecting the onset of a new episode. Categorical expert ratings, such as structured clinical interviews (First et al. 2015), may be advantageous, as they cover two weeks instead of three/four days. However, as categorical ratings fix symptomatology over two weeks, the precise beginning of an upcoming episode and the severity of the episode remain unknown. This is illustrated in Fig. 1b.

**Fig. 1:**
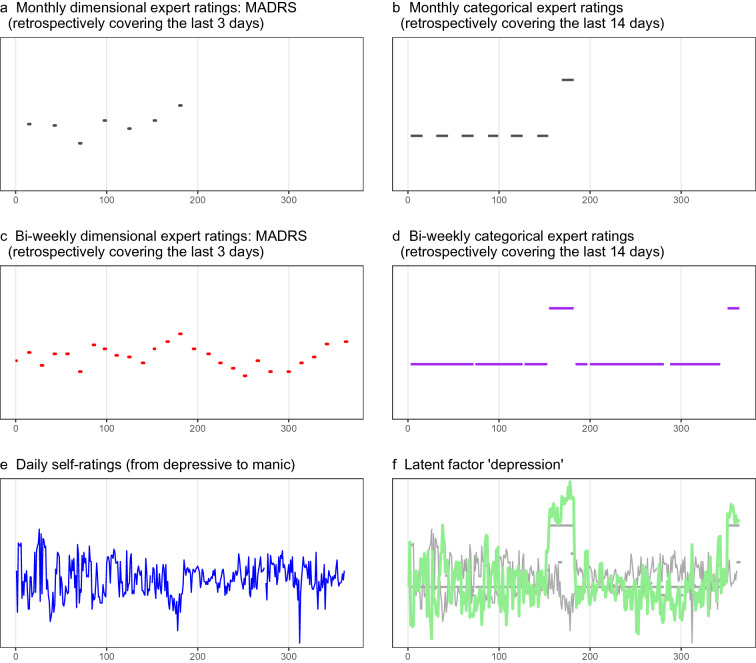
Outcome data (psychopathological status) from patient ID 4458 simulating common assessment strategies (**a**, **b**) and illustrating the actual assessment (**c**–**e**) and the derived latent outcome (**f**). The x-axis represents days, and the y-axis represents standardized values

*The fragmentation of parameters without any integrative analytical strategy * is also problematic. This is the case for both the predictors and the outcomes. In the literature, the number of predictors per study ranged usually between ten and 20, but only when not counting in thousands of used voice parameters (details see Additional file 1 ). Taking multiple outcomes within data sets into account (dimensional and categorical expert and self-ratings for mania and depression) results in hundred possible combinations. This not only raises questions about alpha-error inflation but is also meaningless in a clinical sense. Why should manic communicativeness appear more in phone calls than in text messages? Why should phone calls be more related to dimensional than to categorical expert ratings?

To achieve more consistent findings across studies, we call for (a) longer assessment periods to identify sufficient within-subject variance (“episodes”), (b) frequent and dimensional outcome assessments to reveal timely, accurate, dimensional data indicating symptom severity and (c) integrative analytical strategies to reduce chance findings. Thus, we conducted the BipoSense study, in which we collected biweekly dimensional and categorical expert ratings and daily self-ratings on psychopathological status for 12 months in 29 patients with BD. In addition, we continuously monitored the patients’ digital phenotypes over 12 months by tracking various sensor outputs from smartphones and e-diary ratings. As an integrative analytical strategy, we used latent variables in a structural equation modelling (SEM). Latent variables represent common underlying constructs of observed indicators (Bollen 1989). As an oversimplification, the latent outcome variable “mania” combines the shared variances of dimensional and categorical expert and self-ratings, resulting in one (latent) variable with dimensional values varying on a daily basis.

We hypothesized that (a) substantially increasing the study period and the temporal precision of outcomes is possible, while still achieving excellent compliance, and (b) dimensional and categorical self- and expert ratings, on different time scales, can be combined to two latent psychopathological outcome variables (mania and depression). In addition, we explored whether smartphone parameters can be combined into three different latent digital phenotype domains (activity, sleep, and communicativeness) and whether latent digital phenotype domains are associated with same-day latent psychopathological outcomes.

## Methods

### Study protocol & assessments (BipoSense study)

#### Psychopathological status

All patients were interviewed every two weeks during a 12-month monitoring period (26 assessments per patient in total) and provided additional daily self-reports on their manic-depressive mood. In detail, a trained psychologist provided categorical and dimensional diagnostic instruments alternating in person at the University Hospital Dresden and over the phone. Current affective episodes were determined for the previous two weeks with the SCID-I section A for affective episodes according to DSM-5 (First et al. 2015). (Hypo)manic and depressive symptoms were rated with the German version of the Young Mania Rating Scale (YMRS) (Young et al. 1978), the Bech-Rafaelsen Mania Rating Scale (BRMRS) (Bech et al. 1979), and the MADRS (Montgomery and Asberg 1979), covering the previous three days, each. Reliability and validity of all instruments are excellent (Montgomery and Asberg 1979; Young et al. 1978; Bech et al. 1979). Furthermore, patients answered daily end-of-day diary questions regarding their manic-depressive mood (visual analog scale “depressed” to “elevated”; scale: 0–100), adapted from ChronoRecord (Bauer et al. 2012, 2008).

#### Digital phenotyping

We used the mobile sensing module of movisensXS to track various smartphone sensors (https://www.movisens.com/en/products/movisensxs/). In detail, we monitored the frequency and length of incoming and outgoing phone calls and text messages, number of different call and text contacts, frequency and duration of times the display was on/off, rates of transmitted and received data, travel distances in kilometers, frequency and duration of different activity classes (in vehicle, on bicycle, walking, still, unknown, tilting) and the velocity of movement and number of steps. In addition, we performed end-of-day diaries on sleep (time spent asleep, awake or sleepless in bed over the previous 24 h in 60 min segments) and medications (both questions adapted from ChronoRecord, an extensively validated electronic mood charting system) (Bauer et al. 2008, 2012).

The trial was approved by the IRB of the University Dresden (DE/EKSN38, reference number: 26012014). After having signed informed consent, patients received a study smartphone (optional) and reimbursement of 35 € per month.

### Participants/inclusion and exclusion criteria

Patients were recruited from a specialized outpatient and inpatient clinic for BD at Dresden University Hospital and from patients who contacted the study site directly after seeing articles in print and online media. Out of the 112 patients who initially contacted our study site, 53 patients agreed to be screened for inclusion. Finally, 31 patients were included (e.g. 15 patients did not meet the inclusion criteria, 25 patients did not reply after the first contact, 18 patients reported living too far away, four patients reported technical concerns, while others did not provide reasons for refusal). One person prematurely terminated participation after three weeks due to technical reasons and one person was excluded from the data analysis because the specific smartphone operating system hindered the collection of the mobile sensing data. The final sample consisted of 29 patients.

#### Inclusion criteria

(a) bipolar I and II disorders in full remission at time of enrolment (DSM-5: 296.46; 296.56; 296.89; YMRS score ≤ 12 and MADRS score ≤ 12); (b) ≥ 18 years; (c) ≥ three affective episodes in the last five years, including at least one (hypo)manic episode; and (d) using a smartphone. Exclusion criteria: current substance use disorder (except for tobacco and caffeine); borderline personality disorder; antisocial personality disorder; dementia; organic brain disorders; unstable or insufficiently treated physical illnesses; clinically relevant cardiovascular, neoplastic or cerebrovascular diseases; and kidney or liver disease.

## Statistical analyses

### Selection and modelling of latent psychopathological outcomes

We investigated the factor structure of the three indicators for depression (daily manic-depressive mood ratings, MADRS, and depressive episodes according to the SCID) and mania (daily manic-depressive mood ratings, BRMRS, YMRS, and manic episodes according to the SCID) using SEM (Du Toit 2008; Bollen 1989) Because the indicators were a mix of continuous variables and dichotomous variables on different time scales (current day, last three days, last two weeks), we decided to use a Bayesian estimator for these measurement models, which has been recommended for dichotomous dependent variables in multilevel models (Asparouhov et al. 2018). We used the default (uninformative priors) in Mplus (Asparouhov et al. 2018) with two chains, 10,000 iterations (the first half of which were discarded as burn-in) and a thinning factor of 300. For all analyses obtained using Bayesian estimators (all models involving the latent psychopathological factors), parameters with 95% credible intervals that did not contain zero were considered statistically significant ≠ 0. For all analyses in a frequentist framework (all other models), statistical significance was determined at a level of α < 0.05.

### Selection and modelling of latent digital phenotype predictors

In a stepwise approach, we (1) preselected indicators based on theoretical and conceptual considerations (mostly eliminating redundant variables; for details see Additional file 2); (2) centered all indicators on their person means, and discarded variables that showed only limited variability and/or excessive skewness and/or kurtosis even after transformations (details in Additional file 2: Table S1); and (c) examined the within-person correlations using a fully saturated two-level SEM and discarded variables that were largely redundant (details are provided in Additional file 2: Tables S2–4).

We then conducted a multilevel confirmatory factor analysis using the remaining 13 variables: six indicators of activity (steps, minutes_inVehicle, minutes_onFoot, minutes_still, distance_travelledFast, distance_travelledSlowly), five indicators of communicativeness (phonecalls_out, phonecalls_missed, phonecalls_notReached, total_call_duration, number_dialogue_partners), and two indicators of sleep (hours_asleep and wakeup_time) derived from the e-diary (details see Additional file 2). We estimated a three-factor model on the within-person level (on the between-person level, a fully saturated model was used) using Mplus 8.3 (Asparouhov et al. 2018) and the robust maximum likelihood estimator. One residual variance was estimated to be negative (number_dialogue_partners) and therefore set to zero. Two model fit indices suggested good fit (root mean square error of approximation [RMSEA] = 0.049, Standardized Root Mean Square Residual [SRMR] within = 0.054), while the comparative fit index [CFI] with a value of 0.813 was below the conventional criterion for adequate model fit. Modification indices suggested adding a residual covariance between steps and minutes_onFoot, which seems justified from a conceptual perspective, as both variables cover activity on foot. Adding this residual covariance improved the model fit substantially (RMSEA = 0.029; CFI = 0.934 and SRMR within = 0.044).

### Role of the funding source

The study was funded by a personal university budget of U.E.-P. (KIT). The funding source had no involvement.

## Results

### Patient and monitoring characteristics of the BipoSense study

Patients were, on average, 44 years old (SD = 11.9), and 55% were female. In total, 17 were diagnosed with BD type I, and 12 were diagnosed with type II BD. The reported lifetime numbers of depressive episodes, hypomanic episodes and manic episodes were 7.1 (SD = 5.6), 3.0 (SD = 3.8), and 2.8 (SD = 3.5), respectively. Patients participated for 356 days (SD = 15.6). Compliance was excellent, with rates of 97% for the biweekly diagnostic visits, 99% for mobile sensing data, and 89% for e-diary ratings. In combination with the 12-month assessment period, excellent compliance resulted in 299 days per patient (8678 days in total), including valid expert ratings, e-diary ratings, and mobile sensing data. To put this information into context, we show the biweekly dimensional ratings in Fig. 1c, the biweekly categorical ratings in Fig. 1d, and daily self-ratings in Fig. 1e, again using data from patient ID4458. For the whole sample, 39 affective episodes were diagnosed: 21 depressive (0.7/patient), 15 hypomanic (0.5/patient), and three manic (0.1/patient). According to the DSM-5 criteria (American Psychiatric Association 2013), patients were euthymic on 9509 days, depressed on 731 days, hypomanic on 291 days, and manic on 56 days.

### Modelling latent psychopathological outcomes

We used SEM to combine dimensional and categorical expert and self-ratings into two latent psychopathological outcome variables, one for manic and one for depressive psychopathological status. The probability of scale reduction was 1.003 (depression) and 1.001 (mania), indicating satisfactory model convergence. Table 1 lists the standardized factor loadings for both models; these loadings suggested that combining the dimensional and categorical expert and self-ratings was appropriate. Across both models, two patterns were evident. First, expert ratings showed higher factor loadings than the self-ratings, which means that self-ratings were not able to contribute as much as the expert ratings. This was especially evident for mania, which agrees with the empirical evidence that the level of self-awareness of manic episodes is low (Meyer et al. 2020). Second, the dimensional expert ratings revealed very high loadings, even above the categorical expert ratings using the DSM-5 criteria. This is not surprising, as the categorical, binary outcome only differentiates between presence vs. absence of episodes but cannot measure illness severity. Figure 1f illustrates the latent depression score (green line) combining all ratings into a single-dimensional “depression” variable varying on a daily basis over 12 months. Daily self-ratings were coded from depressed to manic. Accordingly, lower values for self-ratings (blue line in Fig. 1e and the respective grey line in Fig. 1f) correspond to higher depression values for expert ratings.

**Table 1:** Standardized factor loadings of the latent psychopathological outcome for depression (upper panel) and mania (lower panel)

	Factor loading
Latent psychopathological outcome: depression	
MADRS	0.973 [0.918; 0.998]
Manic-depressive mood^1^	− 0.379 [− 0.411; − 0.347]
Depr-SCID^2^	0.848 [0.798; 0.895]
Latent psychopathological outcome: mania	
YMRS	0.879 [0.823; 0.932]
BRMRS	0.908 [0.855; 0.968]
Manic-depressive mood	0.190 [0.146; 0.232]
Man-SCID^2^	0.589 [0.512; 0.661]

95% credible intervals are depicted in square brackets

^1^negative loadings, as high values indicate low depression

^2^0 = no current depressive/manic episode; 1 = current depressive/manic episode

### Modelling latent digital phenotype predictors

**Fig. 2:**
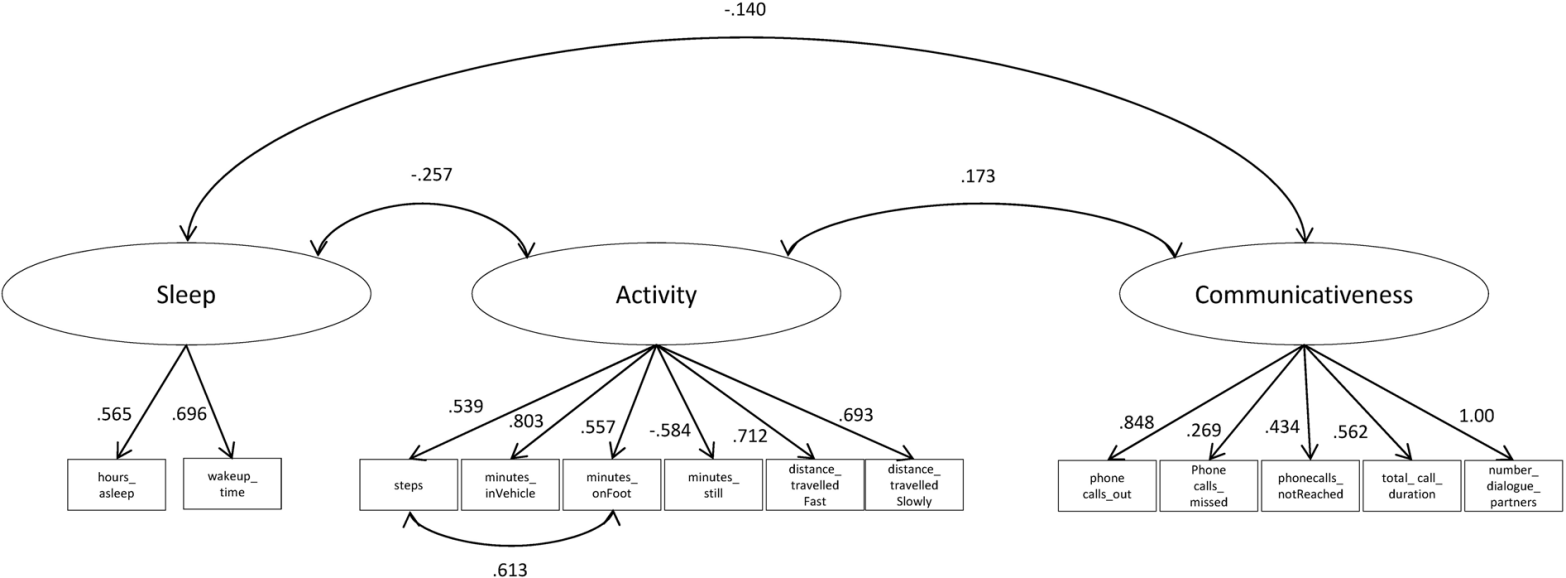
Model with three latent factors for the domains of sleep, activity, and communicativeness, based on 13 smartphone parameters, showing within-subject standardized factor loadings and correlations. All correlations and factor loadings are statistically significant (p < 0.001)

We used multilevel SEM to model one latent digital phenotype predictor for each domain (sleep, activity, communicativeness), as depicted in Fig. 2. The overall model fit was acceptable (RMSEA = 0.029; CFI = 0.934 and SRMR within = 0.044), suggesting empirical evidence for the assumption of underlying common factors (latent variables) for the three domains. All smartphone parameters showed significant positive loadings on their respective domains, with the lowest loadings for predictors with limited variance (such as missed phone calls). Correlations across domains were weak, suggesting that these three factors could be separated and contribute largely independent information.

### Latent digital phenotype predictors related to same-day latent psychopathological outcomes

**Fig. 3:**
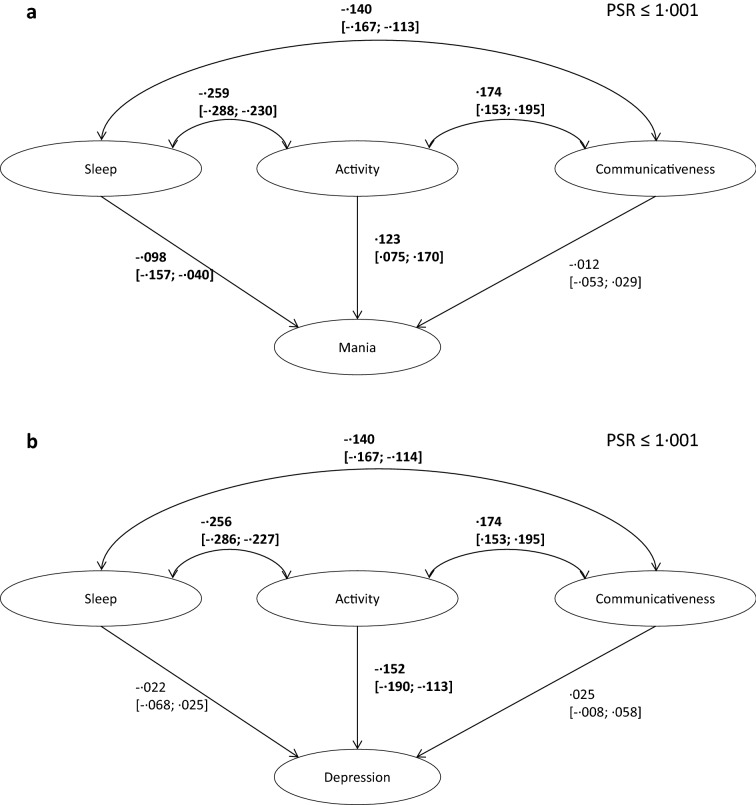
Structural model linking within-person fluctuations in mania (**a**) and depression (**b**) to within-person fluctuations in sleep, activity, and communicativeness. Figure depicts standardized estimates (with associated 95% credible intervals in square brackets). Note that only the within-person associations are depicted

Figure 3a depicts latent mania status, predicted by the three latent domains (zero-order predictor-outcome relations are explicated in Additional file 3). Day-to-day fluctuations in the latent mania status were associated with day-to-day fluctuations in activity (β = 0.123[0.075; 0.170]), indicating that days with more activity were also days with higher levels of mania. Additionally, there was a statistically meaningful effect with regard to sleep (β = − 0.098[− 0.157; − 0.040]), with days with a shorter sleep duration and earlier end of night sleep also being days with higher levels of mania. Communicativeness had no unique effect on mania above and beyond the other predictors (β = − 0.012[− 0.053; 0.029]). Overall, the model explained 3.2% of the daily variability in mania.

Figure 3b depicts the prediction of latent depressive status. Daily fluctuations in latent depressive status were uniquely predicted by same-day activity (β = − 0.152, [− 0.190; − 0.113]) but not by sleep (β = − 0.022[− 0.068; 0.025]) or communicativeness (β = 0.025[− 0.008;0.058]). That is, days with higher than average activity were days with lower depression. The three latent predictors together accounted for 2.2% of the within-person variability in depressive status.

## Discussion

Pioneering work on digital phenotyping in BD has yielded inconsistent findings (Rohani et al. 2018). This is not surprising, as pioneering work on other emerging methods has also yielded inconsistent results, such as the initial work on fMRI or genetics (Tam et al. 2019; Eklund et al. 2016). We propose that short assessment periods, rare outcome assessments, and an extreme fragmentation of parameters without any integrative analytical strategy are the factors driving these inconsistencies.

We choose a 12-months study period and frequent outcome/psychopathological assessments which resulted, in combination with our excellent compliance, in 299 annotated days per patient, which is clearly above benchmark studies (Faurholt-Jepsen et al. 2015a; Faurholt-Jepsen et al. 2019). It is important to mention that sufficient within-subject variance is not only necessary to reveal significant associations, but also to prevent false positive findings (Button et al. 2013).

In the first analytical step, we used multilevel SEM to integrate the categorical expert ratings, dimensional expert ratings, and dimensional self-ratings at varying time intervals. The models converged successfully, and all factor loadings were statistically meaningful, suggesting that it is feasible to construct latent psychopathological outcomes by combining the gold standard categorical expert ratings, daily dynamic information, and fluctuations in severity within and between episodes.

We also integrated smartphone parameters into three latent domains on the predictor level (sleep, activity, and communicativeness). Model fits were good, indicating that smartphone sensor data can meaningfully be combined into these three latent dimensions. In the last exploratory step, we successfully predicted psychopathological status using the three latent digital phenotype predictors. Sleep and activity were statistically meaningfully associated with same-day manic psychopathology, whereas for depression, only activity was statistically significant. This suggests that psychopathological alterations in BD relate to these domains and not only to specific behaviors, which fits nicely to current authoritative systems (American Psychiatric Association 2013). Translated into clinical practice, this denotes, that communicativeness in general is altered in patients with BD, not only specific parameters, such as the number of declined incoming calls.

In both models (mania, depression), the explained variance was modest, namely, 3.2% and 2.2%, respectively. Unfortunately, previous papers rarely reported the amount of explained variance, but in those that did, the amounts were comparable (Gershon et al. 2016). Therefore, our findings are in line with the mixed and contradictory findings of earlier studies (Rohani et al. 2018) and recent randomized controlled trials (RCTs) (Faurholt-Jepsen et al. 2015a,^2019^). However, the generation of a solid data set, covering daily fluctuations in symptom severity, enables to empirically determine: (a) which temporal resolution yields the most reliable estimates, (b) which combination of digital phenotypes best predict psychopathology (“three out of seven” similar to classification systems (American Psychiatric Association 2013), (c) which parameters to integrate as digital phenotypes (given the relation between daily mood ratings and the latent outcome depression in Table 1 might push the idea to use daily mood ratings as digital phenotyping predictor, which would translate to 15.5% explained variance for depression), (d) whether there are nonlinear relations, and (e) how to use individualized prediction models to enable the personalization of medicine (Fisher and Boswell 2016).

Although our study did address preexisting shortcomings, some remaining limitations must be noted. First, even though our data set likely has the most labelled days/patient, and although we only included patients with a high number of previous episodes, the actual number of upcoming episodes was still limited (0.7 depressive episodes/patient; 0.6 (hypo)manic episodes/patient). Simulation studies are needed to estimate if within-subject variance (i.e., few episodes) can be compensated for by number of participants. For treatment studies focused on preventing new episodes, even longer study durations might be warranted, such as 18 months in our currently running RCT (Mühlbauer et al. 2018). Second, it has been speculated that frequent assessments of psychopathological status might hinder new episodes. With our biweekly interviews and daily ratings, our design maximizes this issue. However, we diagnosed more upcoming affective episodes during the 12-month assessment than expected according to the patients’ lifetime histories (estimated 0.3 depressive, 0.1 hypomanic, and 0.1 manic episodes per year and participant, assuming an onset at age 20). Third, we argued that latent constructs should result in more reliable estimates, solving the problem of replication. Even though the model fits were convincing, additional studies are needed to replicate the reported factor structure. Fourth, as in other studies (Faurholt-Jepsen et al. 2015b, 2019; Gershon et al. 2016; Palmius et al. 2017), the selection of parameters was, at least to some extent, based on technology. That is, we used parameters that were easy to track with mobile sensing. Future studies might explore more specific symptoms (such as the fluidity of communication or volume of speech).

To fully leverage the potential of digital phenotyping, we recommend the future studies do the following:

- Ensure sufficient within-subject variance in psychopathological status (patients without manic/depressive episodes do not contribute as much).
- Maximize the number of labelled days.
- Increase the temporal accuracy of the psychopathological status (having precise data on which days the episode began and ended improves the prediction).
- Design a measurement model that includes the severity of psychopathology (with more than presence or absence of an episode).
- Develop measurement models to integrate fragmented variables, thereby limiting the inflation of chance (e.g. using latent variables).

## Supplementary information

**Additional file 1.** Literature screening: digital phenotyping studies in BD.

**Additional file 2.** Parameterization, selection and modelling of latent digital phenotype predictors.

**Additional file 3.** Model selection.

## Data Availability

Data sharing is not applicable at this stage of the study process, as many research questions are not analyzed yet. After final publication of additional research questions, the datasets analysed will be available from the corresponding author on reasonable request.

## Abbreviations

MADRS: Montgomery-Asberg Depression Rating Scale
SEM: Structural equation modelling
YMRS: Young Mania Rating Scale
BRMRS: Bech-Rafaelsen Mania Rating Scale
RMSEA: Root mean square error of approximation
CFI: Comparative fit index
SRMR: Standardized root mean square residual
SD: Standard deviation
RCTs: Randomized controlled trials
