# Supplementary material for: Digital phenotyping: towards replicable findings with comprehensive assessments and integrative models in bipolar disorders
Source: Int J Bipolar Disord. 2020 Nov 17;8:35. doi: 10.1186/s40345-020-00210-4 (PMC7677415; doi:10.1186/s40345-020-00210-4)
Supplement: Supplementary file 1 — Additional file 1. Literature screening: digital phenotyping studies in BD. [file 40345_2020_210_MOESM1_ESM.docx]

**Additional file 1 - Literature screening: digital phenotyping studies in BD**

To get more detailed information about often used methodological features in digital phenotyping studies in BD, we screened the literature to extract information about: i) study period length, ii) assessment frequency (i.e., days between two psychopathological expert assessments), and c) number of predictors per study. To do this we extended the published review from Rohani et al. (2018) by applying their inclusion and exclusion criteria to the 3.5 missing years 2017 to 2020 (literature search performed on 13.08.2020). However, we limited our search to bipolar patients only, resulting in the following search term: *(smartphone OR mobile OR wearable OR “smart phone” OR app OR apps) AND (bipolar) AND (“2017/01/01”[Date–Publication]: “2020/08/13”[Date–Publication]) AND English[Language]*. Our search resulted in 206 publications. Two experts independently reviewed all abstracts and excluded papers: a) studies not assessing patients with bipolar disorder, b) reviews, commentaries or study protocols, c) feasibility studies only reporting feasibility, d) studies not reporting mobile sensing data, and e) studies with just one single expert-rating assessment of psychopathology. This resulted in 4 remaining papers from the years 2017 to 2020.

In addition, two experts independently reviewed all papers with clinical samples from the Rohani et al. (2018) review, covering the years 2007 to 2016. We excluded papers: a) studies not assessing patients with bipolar disorder (i.e. focussing on unipolar depression), thereby excluding: Berle et al., 2010; Dickerson et al., 2011; Doryab et al., 2016; Faurholt-Jepsen et al., 2012, 2015; Hauge et al., 2011; Loprinzi & Mahoney, 2014; Miwa et al., 2007; O´Brien et al., 2016, Todder et al., 2009), b) studies with just one single expert-rating assessment of psychopathology, thereby excluding: Abdullah et al., 2016; Cho et al., 2019; Gonzales et al., 2014; Krane-Gartiser et al., 2014; Palmius et al., 2017; Palmius et al., 2018; St-Armand et al., 2013, as well as c) case studies with just one single participant (Guidi et al., 2015). Accordingly, from the 26 papers with clinical samples Rohani et al. (2018) review, 10 papers remained from the years 2007 to 2016.

Two independent experts read each remaining manuscript (10 manuscripts from 2007 to 2016 as well as 4 manuscripts from 2017 to 2020) and extracted: i) study period length, ii) assessment frequency (i.e. days between two psychopathological expert assessments), and iii) number of predictors per study (i.e. in some cases, the sum of all predictors reported in various manuscripts).

|  | mean | median | modus | range |
| --- | --- | --- | --- | --- |
| study period | 134 days | 87 days | 84 days | 30-365 days |
| assessment frequency | 28.5 days | 21 days | 30 days | 6 – 67,5 days |
| predictors per study | 12.2***** | 12 | 7 | 4-6574 |

********Three papers^4, 11, 12^, were not included when analyzing the mean of ‘predictors per study’, as these papers were based on 6652 different voice parameters, which would bias results on the mean as central tendency.*

Papers finally included in our literature search

1 Alvarez-Lozano J, Osmani V, Mayora O, et al. Tell me your apps and I will tell you your mood: correlation of apps usage with bipolar disorder state. In: Proceedings of the 7th International Conference on PErvasive Technologies Related to Assistive Environments: 1–7.

2 Beiwinkel T, Kindermann S, Maier A, et al. Using smartphones to monitor bipolar disorder symptoms: a pilot study. *JMIR mental health* 2016; **3:** e2.

3 Faurholt-Jepsen M, Busk J, Frost M, et al. Voice analysis as an objective state marker in bipolar disorder. *Translational psychiatry* 2016; **6:** e856-e856.

4 Faurholt-Jepsen M, Busk J, Þórarinsdóttir H, et al. Objective smartphone data as a potential diagnostic marker of bipolar disorder. *Australian & New Zealand Journal of Psychiatry* 2019; **53:** 119–28.

5 Faurholt-Jepsen M, Frost M, Vinberg M, Christensen EM, Bardram JE, Kessing LV. Smartphone data as objective measures of bipolar disorder symptoms. *Psychiatry research* 2014; **217:** 124–27.

6 Faurholt-Jepsen M, Frost M, Christensen EM, Bardram JE, Vinberg M, Kessing LV. The effect of smartphone-based monitoring on illness activity in bipolar disorder: the MONARCA II randomized controlled single-blinded trial. *Psychological medicine* 2020; **50:** 838–48.

7 Faurholt‐Jepsen M, Vinberg M, Frost M, Christensen EM, Bardram JE, Kessing LV. Smartphone data as an electronic biomarker of illness activity in bipolar disorder. *Bipolar disorders* 2015; **17:** 715–28.

8 Faurholt‐Jepsen M, Vinberg M, Frost M, et al. Behavioral activities collected through smartphones and the association with illness activity in bipolar disorder. *International journal of methods in psychiatric research* 2016; **25:** 309–23.

9 Gentili C, Valenza G, Nardelli M, et al. Longitudinal monitoring of heartbeat dynamics predicts mood changes in bipolar patients: A pilot study. *Journal of Affective Disorders* 2017; **209:** 30–38.

10 Gershon A, Ram N, Johnson SL, Harvey AG, Zeitzer JM. Daily actigraphy profiles distinguish depressive and interepisode states in bipolar disorder. *Clinical Psychological Science* 2016; **4:** 641–50.

11 Grünerbl A, Muaremi A, Osmani V, et al. Smartphone-based recognition of states and state changes in bipolar disorder patients. *IEEE Journal of Biomedical and Health Informatics* 2014; **19:** 140–48.

12 Muaremi A, Gravenhorst F, Grünerbl A, Arnrich B, Tröster G. Assessing Bipolar Episodes Using Speech Cues Derived from Phone Calls. In Cipresso P, Matic A, Lopez G. Pervasive Computing Paradigms for Mental Health. Cham: Springer International Publishing, 2014, 103-114.

13 Osmani V, Maxhuni A, Grünerbl A, Lukowicz P, Haring C, Mayora O. Monitoring activity of patients with bipolar disorder using smart phones. In: Proceedings of International Conference on Advances in Mobile Computing & Multimedia: 85–92.

14 Zulueta J, Piscitello A, Rasic M, et al. Predicting mood disturbance severity with mobile phone keystroke metadata: a biaffect digital phenotyping study. *Journal of medical Internet research* 2018; **20:** e241.
