# Supplementary material for: Digital phenotyping: towards replicable findings with comprehensive assessments and integrative models in bipolar disorders
Source: Int J Bipolar Disord. 2020 Nov 17;8:35. doi: 10.1186/s40345-020-00210-4 (PMC7677415; doi:10.1186/s40345-020-00210-4)
Supplement: Supplementary file 2 — Additional file 2. Parameterization, selection and modelling of latent digital phenotype predictors. [file 40345_2020_210_MOESM2_ESM.docx]

**Additional file 2 Parametrization, selection and modelling of latent digital phenotype predictors**

Parametrization of smartphone parameters resulted in a total of 52 variables for the three constructs of interest (activity, sleep, communicativeness) covering e.g., frequency and length of incoming and outgoing phone calls and text messages, number of different call and text contacts, frequency and duration of times the display was on/off, rates of transmitted and received data, daily travel distances in kilometers, frequency and duration of different activity classes (in vehicle, on bicycle, walking, still, unknown, tilting) as well as the velocity of movement and number of steps, all derived from the acceleration sensor of the smartphone (details see Table S1). In a first step, we pre-selected indicators based on theoretical and conceptual considerations, mostly dropping redundant variables. In a second step, we centered all indicators on their person means, and discarded variables that showed only limited variability and / or excessive skewness and / or kurtosis even after transformations (all details in Table S1), resulting in 25 remaining variables. In a third step, we examined the within-person correlations using a fully saturated two-level structural equation model (Mplus 8.3) and dropped additional variables, which were not consistently correlated with the other variables of each domain, were only partially correlated among each other, or were largely redundant. Details are provided in Tables S2-S4. We dropped one of two variables within the activity domain, which were highly correlated (r = .968), suggesting redundancy.

**Table S1. Parametrization and selection of latent digital phenotype predictors**

| **Variable name** | **Variable meaning** | **Retained after Step 1?** | **Reason for exclusion in Step 1** | **Retained after Step 2?** | **Reason for exclusion in Step 2** | **Variable transformed?** | **Retained after Step 3?** |
| --- | --- | --- | --- | --- | --- | --- | --- |
| **Depression / Mania** |  |  |  |  |  |  |  |
| manic-depressive mood | daily rating of manic-depressive mood | Yes |  | Yes |  | No | Yes |
| YMRS | Young Mania Rating Scale | Yes |  | Yes |  | Log-transformed (y = log(x+1)) | Yes |
| BRMRS | Bech Rafaelsen Mania Rating Scale | Yes |  | Yes |  | Log-transformed (y = log(x+1)) | Yes |
| MADRS | Montgomery Asberg Depression Rating Scale | Yes |  | Yes |  | Log-transformed (y = log(x+1)) | Yes |
| depr-SCID | depressive episode according to SCID; dichotomous | Yes |  | Yes |  | No | Yes |
| man-SCID | manic episode according to SCID; dichotomous | Yes |  | Yes |  | No | Yes |
| **Sleep** |  |  |  |  |  |  |  |
| h_awake | number of hours spent awake | No | Redundant with hours_asleep |  |  |  |  |
| hours_asleep | number of hours spent asleep | Yes |  | Yes |  | No | Yes |
| h_sleeples | number of hours spent sleepless | Yes |  | No | Limited variability within individuals |  |  |
| sleep_wake_changes | index for changes between sleep and wake states | Yes |  | Yes |  | Log-transformed (y = log(x+1)) | No |
| wakeup_time | Time of wakeup | Yes |  | Yes |  | No | Yes |
| centroid_sleep | time dividing sleep phase in two equal halves | Yes |  | No | Excessive kurtosis, limited variability within individuals |  |  |
| centroid_inBed | time dividing time in bed in two equal halves | Yes |  | No | Excessive kurtosis, limited variability within individuals |  |  |
| **Communicativeness** |  |  |  |  |  |  |  |
| n_phone | number of phone calls (total) | No | Confound outgoing calls (active) with incoming calls (passive) |  |  |  |  |
| n_phone_in | number of incoming phone calls | No | No active behaviour |  |  |  |  |
| phonecalls_out | number of outgoing phone calls | Yes |  | Yes |  | Log-transformed (y = log(x+1)) | Yes |
| phonecalls_missed | number of incoming missed phone calls | Yes |  | Yes |  | Log-transformed (y = log(x+1)) | Yes |
| phonecalls_notReached | number of outgoing not reached phone calls | Yes |  | Yes |  | Log-transformed (y = log(x+1)) | Yes |
| total call duration | total call duration | Yes |  | Yes |  | Log-transformed (y = log(x+1)) | Yes |
| number of dialogue partners | number of different dialogue partners | Yes |  | Yes |  | Log-transformed (y = log(x+1)) | Yes |
| n_SMS | number of text messages (total) | No | Confound outgoing texts (active) with incoming texts (passive) |  |  |  |  |
| n_SMS_in | number of incoming text messages | No | No active behaviour |  |  |  |  |
| n_SMS_out | number of outgoing text messages | Yes |  | No | Excessive kurtosis |  |  |
| words_SMS | total length of all text messages | No | Confound outgoing texts (active) with incoming texts (passive) |  |  |  |  |
| words_SMS_in | length of incoming text messages | No | No active behaviour |  |  |  |  |
| words_SMS_out | length of outgoing text messages | Yes |  | No | Excessive kurtosis |  |  |
| n_contacts_SMS | number of different conversation partners | Yes |  | Yes |  | Log-transformed (y = log(x+1)) | No |
| **Activity** |  |  |  |  |  |  |  |
| steps | count of steps | Yes |  | Yes |  | Log-transformed (y = log(x+1)) | Yes |
| n_vehicle | frequency of activity class „in vehicle“ | No | Duration deemed more important than frequency |  |  |  |  |
| n_bike | frequency of activity class „on bike“ | No | Duration deemed more important than frequency |  |  |  |  |
| n_foot | frequency of activity class „on foot“ | No | Duration deemed more important than frequency |  |  |  |  |
| n_still | frequency of activity class „still“ | No | Duration deemed more important than frequency |  |  |  |  |
| n_unknown | frequency of activity class „unknown“ | No | Duration deemed more important than frequency |  |  |  |  |
| n_tilting | frequency of activity class „tilting“ | No | Duration deemed more important than frequency |  |  |  |  |
| minutes_in Vehicle | duration of activity class „in vehicle“ | Yes |  | Yes |  | Log-transformed (y = log(x+1)) | Yes |
| min_bike | duration of activity class „on bike“ | Yes |  | No | Excessive kurtosis |  |  |
| minutes_onFoot | duration of activity class „on foot“ | Yes |  | Yes |  | Log-transformed (y = log(x+1)) | Yes |
| minutes_still | duration of activity class „still“ | Yes |  | Yes |  | Inverted, then Log-transformed (y = log(x+1)), then inverted again | Yes |
| min_unknwon | duration of activity class „unknown“ | No | activity class unknown denotes that pattern cannot be assigned to a specific activity |  |  |  |  |
| min_tilting | duration of activity class „tilting“ | No | Tilting is a too short event (<1s) to classify full minutes as tilting |  |  |  |  |
| mean_speed | mean speed of movement | No | Distance (see below) deemed more important than duration |  |  |  |  |
| speed_fast | speed of movement > 30 km/h | No | Distance (see below) deemed more important than duration |  |  |  |  |
| speed_slow | speed of movement < 30 km/h | No | Distance (see below) deemed more important than duration |  |  |  |  |
| distance_km | distance travelled in kilometres | No | Confounds fast and slow movements |  |  |  |  |
| distance_travelledFast | fast (> 30 km/h) distance travelled in kilometres | Yes |  | Yes |  | Removed three outliers (>1500 km), then Log-transformed y = log(x+1) | Yes |
| distance_travelledSlowly | slow (< 30 km/h) distance travelled in kilometres | Yes |  | Yes |  | Log-transformed (y = log(x+1)) | Yes |
| MovAccInt_mg | acceleration in three-dimensional space | Yes |  | Yes |  | No | No |
| h_active | duration of the accelerometers activity (total) | Yes |  | Yes |  | No | No |
| meanV | area under the frequency spectrum curve | No | More or less redundant with MovAccInt_mg |  |  |  |  |
| inaktiv_smartphone | duration with no smartphone activity | No | Redundant with other variables |  |  |  |  |
| display_on_n | frequency of times when display is on | Yes |  | Yes |  | One outlier removed | Yes |
| display_on_min | duration of time when display is on | Yes |  | Yes |  | Log-transformed (y = log(x+1)) | Yes |
| RX_app_KB | amount of data received by the app | No | Duration display on deemed more important as measure for smartphone activity than data traffic |  |  |  |  |
| RX_mobile_MB | amount of mobile received data | No | Duration display on deemed more important as measure for smartphone activity than data traffic |  |  |  |  |
| RX_total_MB | total amount of received data | No | Duration display on deemed more important as measure for smartphone activity than data traffic |  |  |  |  |
| TX_app_KB | amount of data transmitted by the app | No | Duration display on deemed more important as measure for smartphone activity than data traffic |  |  |  |  |
| TX_mobile_MB | amount of mobile transmitted data | No | Duration display on deemed more important as measure for smartphone activity than data traffic |  |  |  |  |
| TX_total_MB | total amount of transmitted data | No | Duration display on deemed more important as measure for smartphone activity than data traffic |  |  |  |  |

**Table S2. Within-person correlations among the potential sleep variables**

|  | sleep_wake_changes | Wakeup_time |
| --- | --- | --- |
| hours_asleep | .192 | .391 |
| sleep_wake_changes |  | -.148 |

**Table S3. Within-person correlations among the potential communication variables**

|  | phonecalls_missed | phonecalls_notReach | total_call_duration | number_dialogue_partners | n_contacts_SMS |
| --- | --- | --- | --- | --- | --- |
| phonecalls_out | .287 | .438 | .391 | .850 | .234 |
| phonecalls_missed |  | .177 | .127 | .274 | .247 |
| phonecalls_notReach |  |  | .171 | .439 | .191 |
| total_call_duration |  |  |  | .563 | .098 |
| number_dialogue_partners |  |  |  |  | .239 |

**Table S4. Within-person correlations among the potential activity variables**

|  | minutes_inVehicle | minutes_onFoot | minutes_still | distance_travelledFast | distance_travelledSlowly | MovAccInt_mg | h_active | display_on_n | display_on_min |
| --- | --- | --- | --- | --- | --- | --- | --- | --- | --- |
| steps | .442 | .729 | -.415 | .291 | .370 | .185 | .177 | .236 | .167 |
| minutes_inVehicle |  | .457 | -.489 | .595 | .509 | .125 | .183 | .183 | .121 |
| minutes_onFoot |  |  | -.459 | .271 | .395 | .176 | .162 | .226 | .150 |
| minutes_still |  |  |  | -.334 | -.358 | -.116 | -.147 | -.160 | -.107 |
| distance_travelledFast |  |  |  |  | .599 | .084 | .134 | .106 | .111 |
| distance_travelledSlowly |  |  |  |  |  | .118 | .147 | .155 | .120 |
| MovAccInt_mg |  |  |  |  |  |  | .968 | .014 | -.041 |
| h_active |  |  |  |  |  |  |  | .014 | -.041 |
| display_on_n |  |  |  |  |  |  |  |  | .460 |
