# Supplementary material for: Digital phenotyping: towards replicable findings with comprehensive assessments and integrative models in bipolar disorders
Source: Int J Bipolar Disord. 2020 Nov 17;8:35. doi: 10.1186/s40345-020-00210-4 (PMC7677415; doi:10.1186/s40345-020-00210-4)
Supplement: Supplementary file 3 — Additional file 3. Model selection. [file 40345_2020_210_MOESM3_ESM.docx]

**Additional file 3 - Model selection**

Before we tested our predictions regarding the prediction of the latent depression and mania factors from the latent sensor factors (sleep, activity, communicativeness) we set up two models in which the latent psychopathology factor (depression or mania) and the three sensor factors (sleep, activity, communicativeness) were correlated. For these models (as well as for the subsequent models), we used the Bayes estimator with the same settings as described in the method section of the manuscript for the measurement models of depression and mania. Results (see Table S5) showed that the within-person correlation of the latent depression factor with the activity factor was statistically significant, r = -.142. In contrast, the association of depression with either sleep, r = -.013, or communicativeness, r = .002, were not statistically meaningful. The latent mania factor was correlated with activity, r = .146, and sleep, r = -.127, but not communicativeness, r = .023.

**Table S5. Within-person correlations among the latent factors.**

|  | Depression | Sleep | Activity |
| --- | --- | --- | --- |
| Sleep | -.013  [-.031; .059] |  |  |
| Activity | -.142  [-.178; -.107] | -.255  [-.284; -.225] |  |
| Communicativeness | .002  [-.031; .035] | -.141  [-.167; -.113] | .174  [.152; .195] |

|  | Mania | Sleep | Activity |
| --- | --- | --- | --- |
| Sleep | -.127  [-.182; -.070] |  |  |
| Activity | .146  [.101; .189] | -.258  [-.287; -.229] |  |
| Communicativeness | .023  [-.017; .063] | -.141  [-.168; -.114] | .174  [.153; .195] |

Note. Table depicts within-person correlations of the latent factors (95% credible intervals in square brackets). Correlations estimated in the model with depression are reported in the upper panel, correlations estimated in the model with mania are reported in the lower panel.
